# Simulation and Analysis of a Silicon Membrane-Supported Beam–Island Diaphragm for Graphene Piezoresistive MEMS Microphones in High-SPL Acoustic Sensing

**DOI:** 10.3390/mi17060719

**Published:** 2026-06-13

**Authors:** Shengsheng Wei, Chunyuan Li, Yipeng Wang, Junqiang Wang, Mengwei Li

**Affiliations:** 1 Shanxi Key Laboratory of Graphene Sensing Materials and Devices, North University of China, Taiyuan 030051, China; sswei@nuc.edu.cn (S.W.);; 2 Academy for Advanced Interdisciplinary Research, North University of China, Taiyuan 030051, China; 3 School of Semiconductors and Physics, North University of China, Taiyuan 030051, China; 4 School of Instrument and Electronics, North University of China, Taiyuan 030051, China

**Keywords:** MEMS microphone, graphene piezoresistor, high SPL, acoustic sensor

## Abstract

High sound pressure level (SPL) acoustic sensing requires miniaturized microphones that can operate under large acoustic loading while maintaining mechanical linearity, sufficient sensing response, and broadband audio frequency behavior. This work targets high-SPL operation and numerically investigates a graphene piezoresistive MEMS microphone based on a membrane-supported beam–island diaphragm. The proposed structure retains a continuous membrane for acoustic load bearing, while the upper beam–island topology redirects deformation-induced strain toward beam root regions where graphene piezoresistors are placed. This design is intended to increase the local strain available for piezoresistive readout without simply relying on larger global diaphragm deflection. Finite-element analysis was used to optimize the diaphragm geometry and evaluate strain enhancement, pressure response linearity, modal behavior, and harmonic response. Under the 170 dB SPL reference condition, the optimized structure increases the peak structural strain from 47.83 με in a thickness-equivalent solid diaphragm to 562.53 με, achieving an approximately 11.8-fold enhancement in local sensing strain while maintaining a highly linear pressure response (R2 > 0.9999). Additionally, the results also show that the sensor exhibits a high first natural frequency of 64.07 kHz and a small response variation of approximately 0.94 dB within the 0–20 kHz target frequency range, indicating excellent dynamic stability and high-fidelity signal transduction characteristics. To connect the structural response with piezoresistive readout, first-order electromechanical output estimation was further performed using representative graphene gauge factors, quarter-bridge readout assumptions, contact resistance correction, and Johnson-noise-limited signal-to-noise ratio estimation. A ±5% geometric tolerance check further indicates that the membrane side length is the most fabrication-sensitive parameter, while the selected design remains generally robust except for reduced linearity margin under positive membrane side-length deviation. These results demonstrate the potential of the proposed graphene-based MEMS microphone for high-SPL broadband acoustic sensing applications in harsh and high-intensity acoustic environments.

## 1. Introduction

High sound pressure level (SPL) acoustic sensing is required in aerospace noise measurement, aeroacoustic testing, turbulent-flow diagnostics, and propulsion-noise characterization [1]. Sensors used in these environments must withstand large pressure amplitudes while maintaining mechanical linearity, sufficient sensing response, broad bandwidth, and overload margin. Early silicon micromachined microphones showed that MEMS devices could be used for aeroacoustic measurements, but they also revealed that sensitivity, dynamic range, linearity, and structural robustness must be balanced under high-SPL excitation [2,3].

Representative MEMS microphone studies have made clear progress in high-SPL acoustic sensing, but they still leave challenges for more severe applications. Sheplak et al. reported a MEMS microphone for aeroacoustic measurements with a linear response up to 155 dB SPL [3]. This work confirmed the value of MEMS microphones in aeroacoustic environments, but the reported level is still below the 170 dB SPL reference condition considered here. Martin et al. developed a dual-backplate micromachined microphone for aeroacoustic measurements, with a dynamic range from a 41 dB/√Hz noise floor to 164 dB SPL [4]. However, its diaphragm–backplate structure depends on a controlled air gap, so large diaphragm motion may cause gap nonlinearity, squeeze-film effects, contact risk, and overload reliability problems. Yan et al. reported an Al_0.8_Sc_0.2_N bimorph microphone with an acoustic overload point of 147 dB SPL and a dynamic range of 107.5 dB [5]. For this type of multilayer diaphragm, improving overload capability usually requires higher stiffness or smaller deformation, which may reduce the available sensing response and bring stress, electrode layout, and bandwidth constraints. Overall, the above studies have enabled high-SPL acoustic signal measurements to varying extents. However, further application in more severe acoustic environments is still restricted by structural linearity, displacement control, overload reliability, resonance margin, and material/readout constraints. Therefore, research on new high-SPL microphone structures and new functional sensing material platforms remains necessary.

Graphene is a promising material platform for MEMS acoustic sensing because it has low areal mass, high Young’s modulus, high intrinsic strength, MEMS-process compatibility, and strain-dependent electrical response [6,7,8]. Graphene-based acoustic devices have also been explored in several forms. Ni et al. reported an ultrathin graphene diaphragm-based Fabry–Pérot acoustic sensor with a frequency response from 5 Hz to 0.8 MHz and tested acoustic pressures up to 114 dB SPL [9]. This work showed that graphene diaphragms can be used for relatively high-pressure acoustic detection, but the device relies on an optical cavity and a suspended diaphragm, making cavity stability, diaphragm linearity, and packaging robustness important concerns under harsher high SPL loading. Zhao et al. reported highly sensitive microphones based on large freestanding reduced graphene oxide membranes, achieving broadband response from 100 Hz to 50 kHz and a signal-to-noise ratio up to 115 dB at 1 kHz [10]. This work further confirms the acoustic potential of graphene-derived membranes; however, the large-area, highly compliant freestanding membrane it employs is mainly designed for ultra-high sensitivity and far-field sound recognition, while high-SPL MEMS microphones require stricter control of diaphragm displacement, linear operating range, and device compactness. Abrahams et al. introduced a graphene squeeze-film microphone based on pressure-induced resonance frequency modulation [11]. This route expands graphene acoustic sensing, but resonance-based operation is less suitable for a flat and linear broadband response under large acoustic excitation. Overall, these studies show that graphene-based acoustic devices have strong potential, but their structures and readout methods still face limitations in displacement control, mechanical linearity, broadband response, and overload margin. This motivates the development of a graphene-based MEMS microphone structure suitable for high-SPL mechanical stability and localized electromechanical readout.

To address this issue, this work numerically investigates a graphene-based MEMS microphone using a membrane-supported beam–island diaphragm. The goal is not to maximize diaphragm displacement, but to guide acoustic pressure-induced strain toward the beam root regions where graphene piezoresistors are placed. The continuous membrane provides an acoustic loading area and mechanical support, while the top beam–island layer redistributes local stiffness and strain. Finite-element analysis was used to optimize the diaphragm geometry and evaluate strain enhancement, pressure response linearity, stress distribution, modal behavior, and harmonic response. The optimized structure achieves strong local strain enhancement under the 170 dB SPL reference load while maintaining controlled global deflection, highly linear pressure response, and sufficient resonance margin above the target frequency range. These results indicate that the membrane-supported beam–island topology provides a feasible structural design route for graphene-based MEMS microphones targeting aero-engine near-field acoustic measurement and other high-SPL sensing scenarios.

## 2. Materials and Methods Working Principle and Methods

### 2.1. Device Architecture and Working Principle

The proposed device is a graphene piezoresistive MEMS microphone based on a membrane-supported beam–island diaphragm. The diaphragm is not a fully through-etched beam–island structure. Instead, the upper surface is patterned into a beam–island topology, while a continuous membrane remains underneath. The continuous membrane provides the acoustic loading area and structural support, whereas the top-patterned beam–island layer redistributes deformation toward the beam root regions.

**Figure 1:**
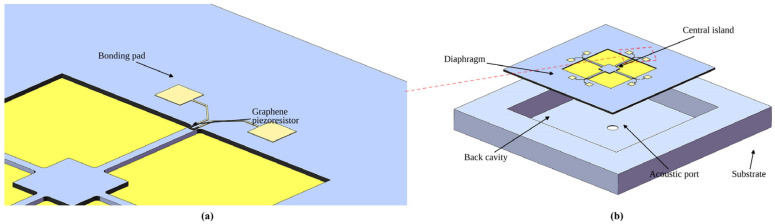
Structural schematic of the proposed graphene piezoresistive MEMS microphone. (**a**) Enlarged view of the graphene piezoresistor and electrode layout near the beam root region. (**b**) Overall view of the membrane-supported beam–island diaphragm, substrate, back cavity, and acoustic port.

The main structural components include the continuous membrane, central island, beam root regions, substrate, back cavity, acoustic port, graphene piezoresistors, metal electrodes, and bonding pads, as shown in Figure 1. Graphene piezoresistors are placed near the beam root regions, where localized strain concentration is expected under acoustic pressure loading. The metal electrodes connect the graphene piezoresistors to the bonding pads for external electrical readout. The present modeling strategy also has limitations. The pressure response analysis is based on static equivalent acoustic pressure loading, and the harmonic response analysis is a simplified structural dynamic simulation. Practical MEMS microphones involve coupled acoustic-structural-fluid interactions, including squeeze-film damping, thermoelastic damping, viscous losses, residual stress, cavity acoustics, packaging effects, and possible nonlinear acoustic behavior under extremely high SPL excitation. Therefore, the present work is intended as a simulation-based structural-electromechanical feasibility study rather than a complete calibrated microphone model.

When acoustic pressure *p* is applied to the diaphragm, the continuous membrane undergoes out-of-plane deformation. The top-patterned beam–island topology modulates the local stiffness distribution and redirects part of the deformation-induced strain toward the beam root regions. The relative resistance change in a graphene piezoresistor can be expressed as

(1) $$\frac{\Delta R}{R}=GF\;\varepsilon$$

where *GF* is the gauge factor of graphene and *ε* is the strain transferred to the piezoresistive region [7,12,13]. Since this work focuses on structural design and finite-element simulation, the reported strain is the finite element-extracted structural strain near the sensing region, rather than an experimentally measured graphene resistance response.

### 2.2. Design Criteria for High-SPL Piezoresistive Microphone

For a high-SPL piezoresistive MEMS microphone, the design objective is not simply to maximize diaphragm displacement [14,15,16,17]. Excessive displacement may increase nonlinear deformation, reduce overload margin, and lower the resonant frequency. Instead, the key objective is to generate high localized strain at the piezoresistive regions while maintaining mechanical linearity and sufficient dynamic bandwidth.

Therefore, the proposed structure was evaluated using the following criteria.

First, the peak structural strain near the beam root sensing regions should be maximized to increase the mechanical signal available for piezoresistive readout. Second, the maximum out-of-plane displacement should remain within the small-deflection regime. The displacement-to-thickness ratio was used as the main linearity indicator:

(2) $$\;\frac{w_{max}}{t_{mem}}\leq 0.2$$

where *w_max_* is the maximum out-of-plane displacement and *t_mem_* is the continuous membrane thickness. Third, the maximum von Mises stress should remain within a mechanically safe range. Finally, the first natural frequency should be sufficiently higher than the target frequency range to maintain a flat pre-resonant response within 0–20 kHz.

In this work, *w_max_*/*t_mem_* < 0.2 was adopted as a conservative small-deflection screening criterion for geometry selection under the 170 dB SPL reference load. This threshold was not treated as a universal failure limit but as a practical design constraint to avoid selecting overly compliant diaphragms with excessive out-of-plane deformation. Physically, when the maximum out-of-plane deflection becomes comparable to the diaphragm thickness, mid-plane stretching introduces geometric (membrane) nonlinearity and the linear small-deflection plate assumption gradually loses validity; keeping *w_max_*/*t_mem_* well below this regime helps preserve a linear pressure–strain relationship and limits nonlinear distortion under high SPL loading.

These criteria were used together to balance strain enhancement, displacement linearity, mechanical safety, and dynamic bandwidth.

### 2.3. Finite-Element Model and Material Properties

A finite-element model of the proposed microphone was established to evaluate the peak structural strain, maximum displacement, stress distribution, natural frequency, and harmonic response under varying diaphragm geometries. The model includes the membrane-supported beam–island diaphragm, a central island, beam root regions, a substrate, a back cavity, and an acoustic port.

All finite-element simulations were performed using ANSYS Workbench 2022 R1 (ANSYS, Inc., Canonsburg, PA, USA). Silicon was selected from the ANSYS Engineering Data library, with a density of ρ = 2330 kg/m^3^. The material coordinate system was aligned with the global coordinate system of the simulation model. Since this work focuses on structural topology comparison rather than crystallographic anisotropy, no additional crystallographic orientation such as [100] or [110] was assigned. The graphene piezoresistors and metal electrodes were included in the structural schematic to indicate the sensing layout. In the mechanical finite-element simulations, the graphene layer was not treated as a load-bearing structural layer because its thickness is negligible compared with that of the silicon diaphragm.

### 2.4. Mesh, Boundary Conditions, and Acoustic Pressure Loading

A locally refined finite-element mesh was applied near the beam root regions, non-through window boundaries, central-island edges, and diaphragm-support interfaces, where relatively large stress and strain gradients were expected. The minimum mesh size was set to 5 μm. A relatively coarser mesh was used in the substrate region to reduce computational cost.

The outer support region of the diaphragm was fixed to represent the clamped boundary condition. A uniform pressure load was applied normal to the diaphragm surface to represent acoustic pressure excitation. The relationship between sound pressure level and acoustic pressure amplitude is

(3) $$p=p_{0}\times 10^{L_{p}/20}$$

where *p*_0_ = 20 μPa is the reference sound pressure and *L*_p_ is the sound pressure level. A sound pressure level of 170 dB SPL corresponds to an acoustic pressure amplitude of approximately 6324 Pa [1].

In the pressure response simulations, six pressure amplitudes were applied: 100, 500, 1000, 2000, 4000, and 10,000 Pa. This pressure range covers the 170 dB SPL-equivalent pressure amplitude. The 6324 Pa value was used as a reference pressure corresponding to 170 dB SPL, rather than as an additional discrete pressure point in the pressure sweep.

A mesh convergence check was further performed under the 170 dB SPL equivalent pressure of 6324 Pa. Three local mesh sizes near the beam root region were compared, including 8 μm, 5 μm, and 3 μm. The peak structural strain, maximum displacement, and maximum von Mises stress were used as convergence indicators. The relative difference between the medium and fine meshes was calculated to evaluate whether the adopted mesh density was sufficient for the subsequent parametric analyses.

### 2.5. Parameter Optimization Strategy

To investigate the effect of diaphragm geometry on high-SPL mechanical response, four key parameters were selected for optimization: *L*_mem_, *L*_island_, *t*_mem_, and *t*_island_, where *L*_mem_ is the diaphragm side length, *L*_island_ is the central-island side length, *t*_mem_ is the continuous membrane thickness, and *t*_island_ is the additional island thickness in the top-patterned region.

For each parameter set, the maximum displacement *w*_max_, peak structural strain *ε*_peak_, maximum von Mises stress *σ*_max_, and displacement-to-thickness ratio *w*_max_/*t*_mem_ were extracted. Specifically, the effects of diaphragm side length, island side length, membrane thickness, and island thickness on strain concentration, displacement linearity, and dynamic bandwidth were systematically investigated.

To avoid treating the design as a single unconstrained maximum-strain problem, the final geometry was selected as a balanced point that satisfies the displacement-linearity constraint while retaining high localized strain. The selected dimensions were *L*_mem_ = 1800 μm, *L*_island_ = 500 μm, *t*_mem_ = 8 μm, and *t*_island_ = 23 μm. The total local material thickness in the reinforced island region is therefore 31 μm. This design should be regarded as a sensitivity-guided and constraint-screened design point rather than a mathematically proven global optimum.

### 2.6. Solid Diaphragm Control and Path-Strain Extraction

To demonstrate the strain-enhancement effect of the proposed topology, the optimized membrane-supported beam–island diaphragm was compared with a thickness-equivalent solid diaphragm control. The control diaphragm thickness was selected as 31 μm, which matches the total local material thickness of the optimized structure:

(4) $$\;t_{\mathrm{control}}=t_{\mathrm{mem}}+t_{\mathrm{island}}=8\;\mu m+23\;\mu m=31\;\mu m$$

Both the solid diaphragm control and the proposed membrane-supported beam–island structure were subjected to the same acoustic pressure loading condition. A representative path was defined across each diaphragm to extract the strain distribution. For the solid diaphragm, the path passes through the central region of the diaphragm. For the proposed structure, the path passes through the beam root regions and the central island. The extracted path strain was used to compare the strain distribution behavior of the two structures and to identify the strain concentration effect near the beam roots.

### 2.7. Pressure Response, Modal, and Harmonic Analyses

The pressure response linearity of the optimized structure was evaluated using static equivalent acoustic pressure simulations. The applied pressure amplitudes were 100, 500, 1000, 2000, 4000, and 10,000 Pa.

For each pressure amplitude, the peak structural strain *ε*_peak_ and maximum displacement *w*_max_ were extracted. Linear regression was performed on the pressure-strain and pressure-displacement relationships. The strain sensitivity *Sε* and displacement sensitivity *S*w were calculated as follows:

(5) $$S_{\varepsilon }=\frac{\Delta \varepsilon_{\mathrm{peak}}}{\Delta p}$$

(6) $$\;S_{w}=\frac{\Delta w_{\max}}{\Delta p}$$

where Δ*ε*_peak_ and Δ*w*_max_ are the changes in peak structural strain and maximum displacement, respectively, and Δ*p* is the change in applied acoustic pressure. The coefficient of determination R^2^ was used to evaluate the linearity of the simulated mechanical response.

Modal analysis was performed to obtain the natural frequencies and mode shapes of the optimized membrane-supported beam–island diaphragm. The first natural frequency, f_1,_ was used as an indicator of resonance margin and dynamic bandwidth.

Harmonic response analysis was further conducted under unit pressure excitation to obtain the frequency-dependent displacement sensitivity. The full frequency response was used to identify the resonance peak, while the response within the 0–20 kHz range was used to evaluate the flatness of the target audio frequency range. In this work, the first three mode shapes are presented.

### 2.8. Electromechanical Readout Estimation

Although the finite-element simulations in this work focus on the mechanical response of the silicon diaphragm, the extracted strain at the beam root sensing region can be used to estimate the electromechanical output of the graphene piezoresistor. For the present device-level estimation, a quarter-bridge configuration is assumed [7,12]. In this configuration, one graphene piezoresistor located near the beam root sensing region serves as the active sensing element, while the other three bridge arms are treated as reference resistors.

Using the graphene piezoresistive relation introduced in Section 2.1, the finite element-extracted strain at the beam root region was used as the mechanical input for the electromechanical readout estimation.

For a practical transferred graphene piezoresistor, the strain experienced by graphene may be lower than the finite element-extracted silicon surface strain due to interfacial strain transfer, graphene wrinkles, transfer-induced defects, passivation, and metal-contact constraints. Therefore, a strain-transfer factor *η* can be introduced, and the effective relative resistance change can be written as [7,12,13]

(7) $$\frac{\Delta R}{R}=\eta \;GF\;\varepsilon$$

where *η* ranges from 0 to 1 and represents the strain-transfer efficiency from the silicon diaphragm surface to the graphene piezoresistor. In the present first-order estimation, *η* = 1 was used to provide an upper-bound electromechanical estimate. A lower *η* would proportionally reduce the estimated voltage sensitivity and SNR. Therefore, graphene transfer quality, interfacial adhesion, passivation design, and electrode layout are critical factors for future experimental implementation.

The strain sensitivity defined in Section 2.7 was used as the mechanical input for the output voltage estimation. From the pressure response simulation, the selected diaphragm provides *S*ε = 0.0890 με/Pa.

For a quarter-bridge biased by an input voltage *V*_in_, the small-signal output voltage sensitivity can be approximated as [7,12]

(8) $$S_{V,q}\approx \frac{V_{\mathrm{in}}}{4}\;GF\;S_{\varepsilon }$$

The output voltage under a given acoustic pressure amplitude *p* can therefore be estimated as


(9) $$V_{\mathrm{out},q}\approx S_{V,q}p$$

In the present estimation, *V*_in_ = 5 V was used, and representative graphene gauge factors of *GF* = 2, 4, and 10 were considered as low, moderate, and relatively high effective parametric values for graphene piezoresistive readout estimation [7,13,18,19]. This estimation is intended to connect the simulated structural strain with the expected electrical output of a quarter-bridge graphene piezoresistive readout, rather than to represent an experimentally calibrated microphone sensitivity.

The influence of graphene-metal contact resistance was also considered [20,21]. If *R*_g_ is the intrinsic resistance of the graphene piezoresistor and *R*_c_ is the contact resistance at each electrode, the effective relative resistance variation becomes

(10) $${\left(\frac{\Delta R}{R}\right)}_{eff}=GF\cdot \varepsilon \cdot \frac{R_{g}}{R_{g}+2R_{c}}$$

Therefore, contact resistance reduces the effective piezoresistive output by the factor *R*_g_/(*R*_g_ + 2 *R*_c_) [20,21]. For example, when *R*_c_/*R*_g_ = 0.1, 0.5, and 1.0, the effective output is reduced to approximately 83.3%, 50.0%, and 33.3% of the ideal value, respectively. This indicates that low-resistance graphene-metal contacts and stable electrode design are important for practical device implementation.

### 2.9. Estimated Noise and SNR Calculation Method

To provide a first-order reference for the noise-limited readout capability, the output-referred thermal noise voltage was estimated using the Johnson–Nyquist thermal noise relation, which is commonly used in piezoresistive sensor noise analysis [12,22]. For a resistor with an equivalent resistance *R*_eq_ over a bandwidth *B* at temperature *T*, the root-mean-square thermal noise voltage is

(11) $$v_{n}=\sqrt{4k_{B}TR_{eq}B}$$

where *k*_B_ is Boltzmann’s constant. The signal-to-noise ratio of the estimated output can be expressed as

(12) $$SNR=20\;log_{10}\left(\frac{V_{out,q}}{v_{n}}\right)$$

The equivalent input-referred noise pressure and its SPL value can be written as

(13) $$p_{n}=\frac{v_{n}}{S_{V,eff}}$$

(14) $$L_{p,n}=20\;log_{10}\left(\frac{p_{n}}{20\;\mu \mathrm{Pa}}\right)$$

In this work, *R*eq = 5 kΩ, *T* = 300 K, and *B* = 20 kHz were used to obtain an order-of-magnitude Johnson-noise-limited estimate. This simplified calculation does not include amplifier noise, 1/f noise, contact resistance fluctuation, temperature drift, acoustic packaging noise, or environmental interference; therefore, it represents an optimistic lower-bound noise estimate rather than a complete microphone noise model.

## 3. Results

### 3.1. Strain Concentration Mechanism Compared with a Solid Diaphragm

**Figure 2:**
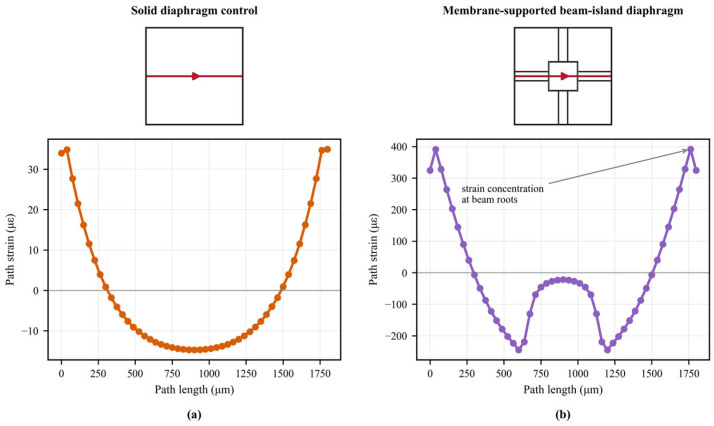
Path-strain distribution comparison between the solid diaphragm control and the proposed membrane-supported beam–island diaphragm. (**a**) Solid diaphragm control. (**b**) Membrane-supported beam–island diaphragm. The upper schematics indicate the representative strain-extraction paths.

To clarify the effect of the membrane-supported beam–island topology, the 31 μm solid diaphragm control was compared with the proposed diaphragm. Figure 2 compares the path-strain distributions of the two structures under the same acoustic pressure loading condition. The solid diaphragm control exhibits a relatively smooth strain distribution along the representative path, with a maximum path strain of 47.83 με. The strain is distributed over a broad region rather than concentrated at a clearly defined sensing location.

In contrast, the proposed membrane-supported beam–island diaphragm shows pronounced strain concentration near the beam root regions. The path strain reaches 562.53 με near the beam root sensing region, while the strain around the central island remains relatively low. This indicates that the top-patterned beam–island topology redirects deformation-induced strain toward localized beam root regions, which are suitable positions for graphene piezoresistors. Compared with the solid diaphragm control, the optimized structure provides approximately 11.8 times higher peak structural strain.

### 3.2. Single-Parameter Parametric Analysis

**Figure 3:**
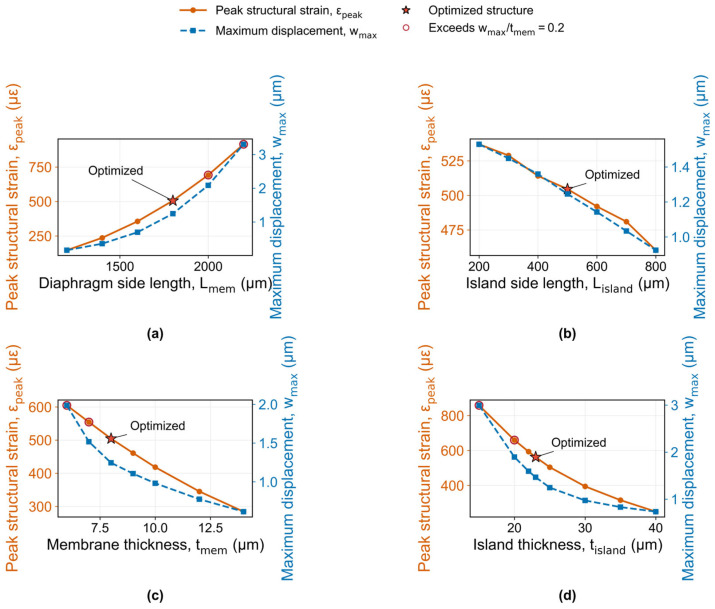
Effects of single geometric parameters on peak structural strain and maximum displacement. (**a**) Diaphragm side length. (**b**) Island side length. (**c**) Membrane thickness. (**d**) Island thickness.

Figure 3 shows the effects of four geometric parameters on peak structural strain and maximum displacement: diaphragm side length *L*_mem_, island side length *L*_island_, membrane thickness *t*_mem_, and island thickness *t*_island_. These parameters directly affect the compliance of the continuous membrane, the stiffness of the top-patterned beam–island topology, and the strain level delivered to the beam root sensing regions.

As *L*_mem_ increases from 1200 to 2200 μm, both peak structural strain and maximum displacement increase because a larger diaphragm provides higher mechanical compliance under the same pressure load. The strain increase is beneficial for piezoresistive readout, but excessive displacement may reduce the linear operating margin. Therefore, *L*_mem_ = 1800 μm was selected as a balanced design point.

Increasing *L*_island_ from 200 to 800 μm reduces both maximum displacement and peak structural strain. A larger central island increases the effective stiffness of the patterned diaphragm and suppresses global deformation, which improves displacement control but reduces the strain transferred to the beam root regions. The selected value of *L*_island_ = 500 μm provides a compromise between strain concentration and structural stiffness.

Increasing *t*_mem_ increases the stiffness of the continuous membrane and consequently decreases both displacement and peak structural strain. Thin membranes such as *t*_mem_ = 6 μm and 7 μm provide higher strain, but their displacement-to-thickness ratios exceed the adopted linearity criterion. The membrane thickness of 8 μm was therefore selected to maintain *w*_max_/*t*_mem_ below 0.2 while retaining a high strain response.

The island thickness *t*_island_ controls the local stiffness of the top-patterned beam–island region. Increasing *t*_island_ from 15 to 40 μm progressively reduces displacement and strain because the central island and beam root regions become stiffer. Very thin island regions produce high strain but also excessive displacement. The selected island thickness of 23 μm satisfies the displacement criterion while maintaining strong strain concentration.

### 3.3. Coupled Parameter Verification and Pareto-Type Design Tradeoff

**Figure 4:**
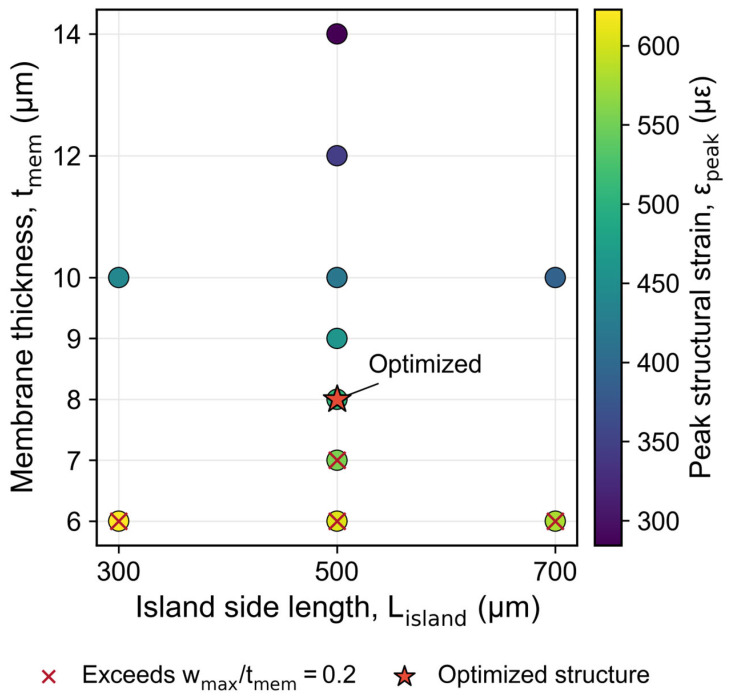
Coupled verification of island side length and membrane thickness. The color map indicates peak structural strain, and red crosses indicate designs exceeding the displacement-to-thickness limit.

Although single-parameter sweeps are useful for identifying the influence of each geometric parameter, the mechanical response of the microphone is determined by coupled parameter interactions. Figure 4 evaluates the combined influence of island side length and membrane thickness. The optimized design lies in a region that provides high peak structural strain while satisfying the displacement-to-thickness criterion. Designs with excessive displacement are excluded because they are more likely to enter a nonlinear deformation regime under high SPL loading.

**Figure 5:**
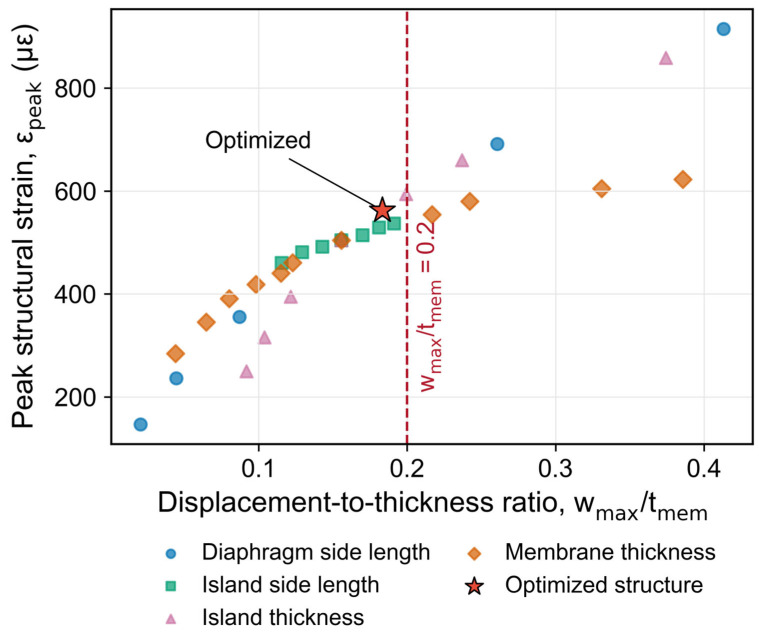
Design tradeoff between peak structural strain and displacement-to-thickness ratio. The dashed vertical line represents the small-deflection threshold *w*_max_/*t*_mem_ = 0.2.

Figure 5 summarizes the design tradeoff between peak structural strain and displacement-to-thickness ratio [17]. In general, higher strain can be obtained by increasing diaphragm compliance, but this also increases *w*_max_/*t*_mem_. Therefore, the design target is not simply the highest strain point, but a balanced point that provides high local strain while remaining below the adopted small-deflection threshold. The optimized geometry achieves this balance by locating the design point close to the high-strain region while maintaining *w*_max_/*t*_mem_ < 0.2.

To provide a semi-quantitative basis for the design selection, a normalized sensitivity analysis was carried out using the single-parameter sweep data. For each geometric parameter *p* and response *Y*, where *Y* represents either the peak structural strain *ε*_peak_ or the maximum displacement *w*_max_, a range-based normalized sensitivity index was defined as

(15) $$S_{Y,p}=\left|\frac{\Delta Y/Y_{ref}}{\Delta p/p_{ref}}\right|$$

Here, Δ*Y* and Δ*p* are the full variations across each single-parameter sweep, while *Y*_ref_ and pref are taken from the final selected design (*ε*_peak_ = 562.53 με, *w*_max_ = 1.466 μm, *L*_mem_ = 1800 μm, *L*_island_ = 500 μm, *t*_mem_ = 8 μm, *t*_island_ = 23 μm). Because the common sweep center in the dataset uses *t*_island_ = 25 μm, this point was treated only as a representative single-parameter sweep baseline rather than as the selected design. The resulting indices show that *L*_mem_ has the strongest influence on both peak strain and displacement (*S*ε ≈ 2.46, *S*w ≈ 3.86). The island thickness also shows a clear influence (*S*ε ≈ 1.00, *S*w ≈ 1.42), followed by the membrane thickness (*S*ε ≈ 0.57, *S*w ≈ 0.93), whereas the island side length has the weakest influence (*S*ε ≈ 0.11, *S*w ≈ 0.34). These results indicate that *L*_mem_ is the dominant parameter governing diaphragm compliance, while *t*_mem_ also contributes to compliance control. In contrast, *L*_island_ and *t*_island_ mainly adjust the local stiffness redistribution of the top-patterned beam–island region and the redirection of deformation-induced strain toward the beam root sensing regions. Accordingly, the selected design should be interpreted as a sensitivity-guided and constraint-screened balanced design point rather than a global mathematical optimum.

A mesh convergence study was conducted under the 170 dB SPL equivalent pressure of 6324 Pa to verify the numerical stability of the optimized structure. Three local mesh sizes near the beam root region were compared. As shown in Table 1, the peak structural strain, maximum displacement, and maximum von Mises stress were used as convergence indicators. Compared with the fine mesh, the medium mesh shows a relative difference of 1.13% in peak structural strain, 0.26% in maximum displacement, and 2.29% in maximum von Mises stress. These differences are sufficiently small for the purpose of parametric design comparison, indicating that the adopted 5 μm local mesh provides adequate numerical accuracy for the subsequent simulations.

The selected geometry is therefore not the point with the maximum strain alone. Instead, it is a constraint-screened tradeoff point that provides relatively high *ε*_peak_ while maintaining *w*_max_/*t*_mem_ below the adopted small-deflection threshold. This Pareto-type interpretation avoids selecting overly compliant designs that produce higher strain but may suffer from reduced linearity margin and lower resonance frequency. Therefore, the selected design is better described as a balanced high-SPL structural design rather than a purely strain-maximized diaphragm.

### 3.4. Pressure Amplitude Response Linearity

**Figure 6:**
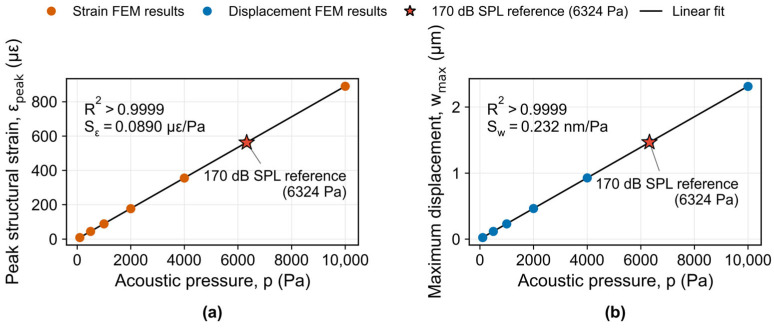
Simulated pressure amplitude response of the optimized structure. (**a**) Pressure-strain response. (**b**) Pressure-displacement response. The star marks the 170 dB SPL reference pressure.

The pressure amplitude response of the optimized structure was evaluated under static equivalent acoustic pressure loads of 100, 500, 1000, 2000, 4000, and 10,000 Pa. This pressure range covers the 170 dB SPL-equivalent pressure amplitude of 6324 Pa, which is used as the reference point in Figure 6.

The peak structural strain increases almost linearly with applied acoustic pressure. Linear fitting gives a structural strain sensitivity of *S*ε = 0.0890 με/Pa with R^2^ > 0.9999. Similarly, the maximum displacement increases linearly with pressure, with a displacement sensitivity of *S*w = 0.232 nm/Pa and *R*^2^ > 0.9999. At the 170 dB SPL reference pressure, the optimized structure shows a maximum displacement of approximately 1.466 μm and a peak structural strain of 562.53 με.

It should be emphasized that the pressure amplitude response analysis is based on static equivalent pressure loading. Therefore, these results describe the structural linearity of the diaphragm under acoustic pressure amplitude loading rather than a complete calibrated microphone output.

### 3.5. Modal Characteristics

**Figure 7:**
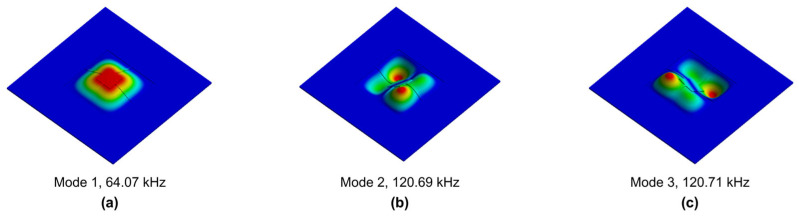
First three simulated mode shapes of the optimized membrane-supported beam–island diaphragm. (**a**) Mode 1 at 64.07 kHz. (**b**) Mode 2 at 120.69 kHz. (**c**) Mode 3 at 120.71 kHz.

Modal analysis was performed to evaluate the dynamic behavior of the optimized membrane-supported beam–island diaphragm. Figure 7 shows the first three mode shapes of the optimized structure. The corresponding natural frequencies are 64.07, 120.69, and 120.71 kHz.

The first mode is mainly associated with the out-of-plane deformation of the diaphragm and central island. Since the first natural frequency is higher than 20 kHz, the diaphragm is expected to operate in a pre-resonant region over the 0–20 kHz target frequency range. The second and third modes are close in frequency and represent higher-order deformation patterns associated with the symmetry of the beam–island topology.

The modal participation results further confirm the dominance of the first pressure-driven mode. In the manuscript coordinate convention, the Z direction denotes the out-of-plane diaphragm-normal direction of the applied acoustic pressure. The first mode has the largest Z-direction modal participation and accounts for approximately 86.1% of the cumulative extracted effective modal mass in the diaphragm-normal direction. In contrast, the second and third modes show negligible participation in this direction, indicating that their contribution to the uniform pressure-driven response is limited. The next noticeable diaphragm-normal modal contribution appears at a higher mode above 160 kHz, which is far beyond the 0–20 kHz target frequency range. Therefore, mode coupling is not expected to significantly affect the pre-resonant audio-band mechanical response of the selected design.

### 3.6. Harmonic Response

**Figure 8:**
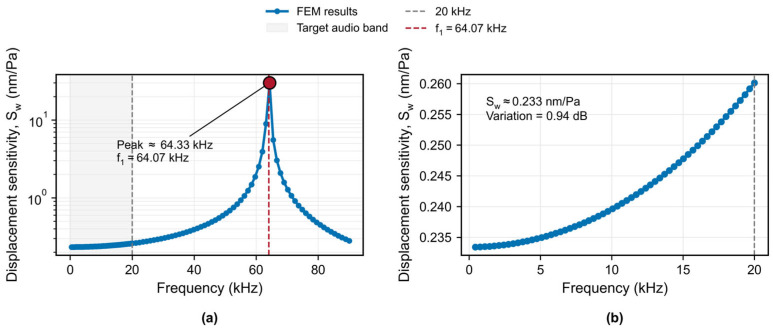
Harmonic response of the optimized structure under unit pressure excitation. (**a**) Full-frequency displacement sensitivity response. (**b**) Enlarged response within the target 0–20 kHz frequency range.

The frequency-dependent displacement sensitivity of the optimized structure was evaluated using harmonic response analysis under unit pressure excitation. Figure 8a shows the full harmonic response, where a resonance peak appears near 64.33 kHz. This peak is close to the first natural frequency obtained from modal analysis, confirming the consistency between the modal and harmonic simulations.

Figure 8b shows the displacement sensitivity within the 0–20 kHz target frequency range. The simulated response remains relatively flat over this frequency range, with an average displacement sensitivity of approximately 0.233 nm/Pa. The variation within 0–20 kHz is approximately 0.94 dB. This indicates that the optimized diaphragm provides a nearly flat pre-resonant mechanical response over the target acoustic band while retaining a sufficient resonance margin.

### 3.7. Estimated Electrical Output Based on Quarter-Bridge Graphene Piezoresistive Readout

To further connect the simulated structural strain with the expected piezoresistive readout, the electrical output was estimated using the strain sensitivity extracted from the finite-element pressure response results. The optimized diaphragm provides a strain sensitivity of 0.0890 µε/Pa. Assuming V_in_ = 5 V, the estimated voltage sensitivity of a quarter-bridge readout was calculated using representative graphene gauge factors of *GF* = 2, 4, and 10. The *GF* values of 2, 4, and 10 were selected to represent low, moderate, and relatively high effective graphene piezoresistive responses for parametric readout estimation, rather than calibrated material properties of the present device [7,13,18,19]. Because the first-order calculation uses η = 1, the values in Table 2 represent upper-bound estimates with ideal strain transfer from the silicon surface to the graphene piezoresistor. If η is lower than 1 in a fabricated device, the voltage sensitivity and output voltage would decrease approximately in proportion to η.

Table 2 summarizes the estimated voltage sensitivity and the corresponding output voltage at the 170 dB SPL reference pressure of 6324 Pa. For *GF* = 2, the estimated voltage sensitivity is 0.223 µV/Pa, corresponding to an output voltage of approximately 1.41 mV at 170 dB SPL. When higher effective gauge factors are considered, the estimated output increases proportionally. For example, when *GF* = 10, the quarter-bridge output sensitivity reaches approximately 1.11 µV/Pa, corresponding to 7.04 mV under the 170 dB SPL reference condition.

These results indicate that the local strain enhancement achieved by the membrane-supported beam–island diaphragm can be translated into a measurable quarter-bridge piezoresistive output under reasonable graphene gauge-factor assumptions [7,12,13]. However, the actual electrical sensitivity of a fabricated device will depend on graphene quality, transfer-induced defects, electrode contact resistance, residual stress, passivation, bridge layout, thermal drift, and packaging. Therefore, the present electrical estimation is intended to bridge the finite-element mechanical results and expected piezoresistive readout, rather than replace experimental calibration.

### 3.8. Estimated Noise and Signal-to-Noise Ratio

A first-order thermal noise-limited SNR estimation was further performed to evaluate whether the estimated quarter-bridge output is within a measurable range [12,22]. Assuming *T* = 300 K, *B* = 20 kHz, and a nominal bridge resistance of *R* = 5 kΩ, the output-referred thermal noise voltage is calculated to be approximately 1.29 µV rms.

Table 3 summarizes the estimated equivalent input pressure noise and SNR under the 170 dB SPL reference pressure. For *GF* = 2, the estimated signal voltage at 170 dB SPL is 1.41 mV, which gives a thermal -noise-limited SNR of approximately 60.8 dB. For *GF* = 4 and *GF* = 10, the estimated SNR increases to approximately 66.8 dB and 74.8 dB, respectively.

These results suggest that the estimated quarter-bridge output is sufficiently larger than the thermal noise level under the high-SPL reference condition. Nevertheless, this estimation does not include graphene 1/f noise, amplifier input noise, contact resistance fluctuation, thermal drift, packaging-induced noise, or acoustic calibration uncertainty. Because the estimated output voltage scales approximately with the strain-transfer factor *η*, non-ideal strain transfer would reduce the estimated voltage sensitivity and SNR. For example, *η* = 0.5 would reduce the estimated output by 50% and decrease the SNR by approximately 6 dB. Therefore, the reported SNR values should be interpreted as first-order thermal noise-limited estimates rather than experimental microphone noise specifications.

### 3.9. Benchmarking with Representative High-SPL MEMS and Graphene-Based Microphones

To further clarify the positioning of the proposed design, representative high-SPL MEMS microphones and graphene-based acoustic devices were compared in Table 4. Rather than emphasizing absolute sensitivity alone, the present design focuses on high-SPL structural stability, localized graphene piezoresistive strain enhancement, controlled diaphragm deflection, and pre-resonant audio-band mechanical response. Therefore, the comparison highlights the structural and readout positioning of this simulation-stage design relative to previously reported MEMS and graphene-based acoustic devices. Because the reported metrics and device dimensions vary among different studies, the comparison focuses on directly comparable parameters, including SPL-related capability, sensitivity or output, bandwidth or resonance, sensing mechanism, and structural/readout positioning. When diaphragm dimensions or fabrication complexity were not consistently reported in the original studies, these aspects were summarized qualitatively in the positioning column rather than listed as separate quantitative columns. The values for this work are obtained from finite-element simulation and first-order electromechanical estimation rather than experimental microphone calibration.

### 3.10. Fabrication Tolerance Check

To assess the robustness of the selected design against manufacturing deviations, an additional finite-element tolerance analysis was performed. Each key geometric parameter was perturbed by ±5% around its nominal value, while the remaining parameters were kept at the selected design. All cases were evaluated under the 170 dB SPL equivalent pressure of 6324 Pa with geometric nonlinearity enabled, and the modal analysis was repeated for each perturbed geometry. Table 5 summarizes the resulting relative changes in peak structural strain (*ε*_peak_), maximum displacement (*w*_max_), maximum von Mises stress (*σ*_max_), and the first natural frequency (f1) with respect to the selected design.

The tolerance analysis indicates that the membrane side length *L*_mem_ is the most critical dimension: a +5% deviation increases *w*_max_ by about 36.65% and *ε*_peak_ by about 18.19%, and lowers f1 by about 7.77%. The island thickness *t*_island_ is the next most influential parameter, whereas the island side length *L*_island_ has the weakest effect, with strain variations below 1% under ±5% deviation. This ranking is consistent with the normalized sensitivity analysis in Section 3.3. The selected design preserves the same overall response trend under representative ±5% deviations; however, a positive deviation of *L*_mem_, or a negative deviation of *t*_mem_ or *t*_island_, may bring *w*_max_/*t*_mem_ close to or beyond the adopted small-deflection criterion of 0.2 (for example, 0.250 for *L*_mem_ +5%), which reduces the displacement-linearity margin. Therefore, in practical fabrication, the membrane side length and the key thicknesses should be prioritized for dimensional control.

## 4. Discussion

The simulation results demonstrate that the membrane-supported beam–island diaphragm can enhance local sensing strain without relying solely on large global diaphragm deflection. Compared with the solid diaphragm control, the optimized topology increases peak structural strain by 11.8 times. This improvement originates from stiffness redistribution in the top-patterned beam–island region, which drives strain toward the beam root sensing locations.

The proposed design also involves a clear tradeoff. Increasing diaphragm compliance improves strain response, but it also increases displacement and may reduce resonance frequency. The optimized structure balances these competing requirements by selecting a geometry that provides high beam root strain, acceptable displacement, moderate stress, and a first natural frequency above 60 kHz. Similar structure-performance relationships have also been reported in MEMS microphone studies involving diaphragm topology, support conditions, and acoustic-overload-oriented structural design, supporting the broader idea that local stiffness modulation can improve sensitivity, reliability, and dynamic response [23,24,25]. Broader studies on MEMS microphones also show that device performance is strongly affected by diaphragm design, air-gap configuration, nonlinear distortion, miniaturization, film stress, and readout constraints [26,27,28,29,30]. In addition, the actual electrical sensitivity of a graphene piezoresistive microphone will also depend on elastic modulus, residual stress, the number of graphene layers and others [7,31,32,33].

The elastic modulus of graphene is a critical material parameter that influences local stress and strain distribution at the beam root sensing regions. Pristine single-layer graphene possesses a theoretical Young’s modulus of approximately 1 TPa; however, CVD-grown graphene used in practical MEMS devices typically exhibits lower effective moduli due to polycrystalline grain boundaries, point defects, and wrinkles introduced during synthesis and transfer. Such deviations from ideal elastic properties may shift the local strain values at the piezoresistor locations and should be accounted for when translating FEM-predicted strains into expected piezoresistive outputs [8,13].

Beyond elastic modulus, residual stress in the graphene layer represents another important influencing factor. Residual stress is commonly introduced by thermal mismatch during CVD growth or by mechanical deformation during wet transfer onto the silicon diaphragm, superimposing a pre-stress state onto the sensing regions. Tensile residual stress tends to stiffen the local mechanical response and reduce the effective strain under acoustic loading, whereas compressive residual stress may introduce a buckling risk in thin suspended membrane structures. Both scenarios can lead to discrepancies between idealized simulation predictions and the actual device behavior [7,13,33].

The number of graphene layers further affects the balance between electrical and mechanical performance of the piezoresistive element. Single-layer graphene offers the highest intrinsic gauge factor, which is favorable for maximizing sensitivity; however, few-layer graphene provides improved electrical conductivity and greater process robustness, representing a practical trade-off that must be considered in the transition from simulation to physical device realization [19].

**Figure 9:**
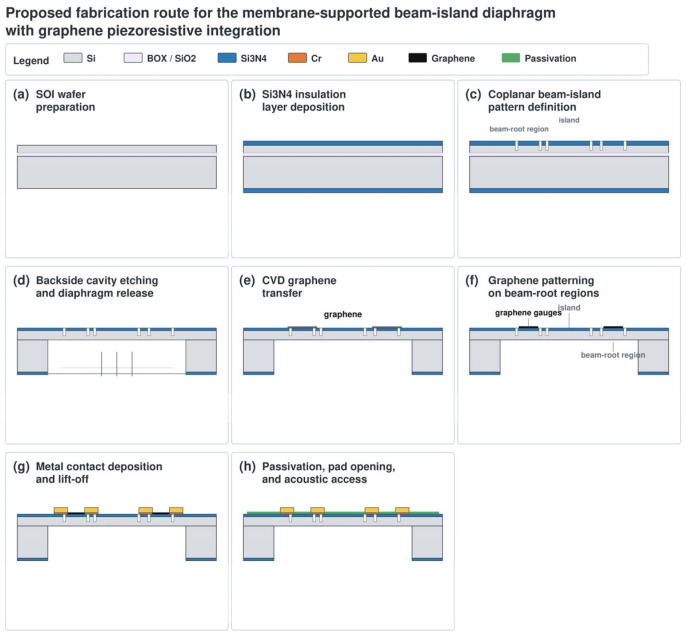
Proposed fabrication route for the SOI sensing chip with graphene piezoresistive integration. (**a**) SOI wafer preparation. (**b**) Si_3_N_4_ insulation layer deposition. (**c**) Coplanar beam–island pattern definition. (**d**) Backside cavity etching and diaphragm release. (**e**) CVD graphene transfer. (**f**) Graphene patterning on the beam root sensing regions. (**g**) Metal contact deposition and lift-off. (**h**) Passivation, pad opening, and acoustic access. This schematic is intended to demonstrate the fabrication feasibility of the SOI sensing chip and graphene integration process rather than a completed experimental realization.

**Figure 10:**
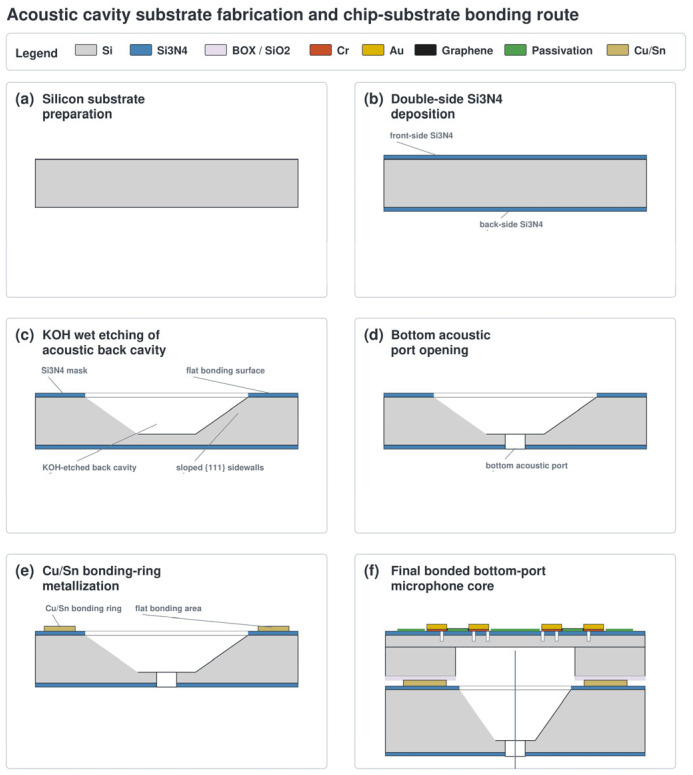
Proposed acoustic cavity substrate fabrication and chip–substrate bonding route for the bottom-port graphene piezoresistive MEMS microphone. (**a**) Silicon substrate preparation. (**b**) Double-sided Si_3_N_4_ deposition for KOH/DRIE masking. (**c**) KOH wet etching of the silicon substrate to form a sloped-wall acoustic back cavity while retaining a flat peripheral bonding surface. (**d**) Backside DRIE opening of the bottom acoustic port connected to the back cavity. (**e**) Cu/Sn bonding-ring metallization on the flat peripheral bonding area. (**f**) Final bonded microphone core, in which the SOI sensing chip is bonded to the acoustic cavity substrate. This schematic is intended to demonstrate the fabrication feasibility of the acoustic cavity substrate and chip–substrate bonding route rather than a completed experimental realization.

While the present study focuses on structural design and FEM simulation, the proposed device concept is compatible with established SOI-MEMS, silicon micromachining, wafer/chip bonding, and graphene integration processes. A practical implementation can be divided into two parallel fabrication routes. The SOI sensing-chip and graphene integration route is illustrated in Figure 9, and the acoustic cavity substrate and chip–substrate bonding route is shown in Figure 10. First, the SOI sensing chip is prepared using the device silicon layer to define the diaphragm thickness. After double-sided Si_3_N_4_ insulation deposition, the membrane-supported beam–island topology can be formed by front-side lithography and timed partial deep reactive ion etching (DRIE), while backside DRIE and BOX removal release the suspended diaphragm region. CVD-grown graphene can then be transferred onto the sensing side, patterned into piezoresistive elements near the beam root strain concentration regions, contacted by Cr/Au electrodes and bonding pads, and protected by an ALD Al_2_O_3_ passivation layer with pad openings. Second, a separate silicon acoustic cavity substrate can be prepared to provide the back cavity, bottom acoustic port, and bonding support. In this substrate route, double-sided Si_3_N_4_ can be used as an etching mask, KOH wet etching can define a sloped-wall acoustic back cavity, backside DRIE can open the bottom acoustic port, and Cu/Sn metallization can form a peripheral bonding ring on the flat bonding surface. The completed SOI sensing chip can then be bonded to the acoustic cavity substrate, forming a bottom-port microphone core.

To further complete the device-level microphone architecture, an acoustic cavity substrate and chip–substrate bonding route are also considered. In this configuration, the SOI sensing chip provides the membrane-supported beam–island diaphragm and graphene piezoresistive readout structure, whereas the separately fabricated silicon cavity substrate provides the acoustic back volume, bottom acoustic port, and mechanical bonding support. The proposed substrate process and final bonded microphone core are schematically shown in Figure 10.

This fabrication outline confirms that the beam–island diaphragm is a manufacturable device concept grounded in SOI-MEMS and graphene processing technologies. Experimental fabrication and characterization are planned as the next phase of this research, with the present FEM results serving as the design basis.

## 5. Conclusions

This work proposed and numerically investigated a graphene piezoresistive MEMS microphone based on a membrane-supported beam–island diaphragm for high-SPL acoustic sensing. In this structure, the continuous underlying membrane and the top-patterned beam–island layer separate the acoustic loading function from the strain-localization function within a single diaphragm architecture. Finite-element optimization showed that, compared with a thickness-equivalent 31 μm solid-diaphragm control, the optimized geometry increases the peak strain at the beam root sensing region from 47.83 με to 562.53 με, corresponding to an approximately 11.8-fold enhancement. Meanwhile, the pressure-strain and pressure-displacement responses remain highly linear with R^2^ > 0.9999, the first natural frequency reaches 64.07 kHz, and the mechanical response variation within 0–20 kHz is approximately 0.94 dB. The pressure amplitude sweep further indicates that the optimized structure maintains a linear mechanical response up to 10 kPa, corresponding to approximately 174 dB SPL, providing additional margin beyond the 170 dB SPL reference condition. These simulation results indicate that strong localized piezoresistive strain can be obtained without relying on large diaphragm deflection, which is important for maintaining mechanical linearity, overload margin, and audio-band flatness under high-SPL-equivalent pressure loading. Overall, the proposed membrane-supported beam–island diaphragm provides a structural design route for graphene piezoresistive MEMS microphones targeting aero-engine near-field acoustic measurement and other high-SPL environments where high sensitivity, mechanical linearity, and broadband response are required simultaneously. It should be noted that the above conclusions are drawn from numerical simulation, and experimental fabrication and characterization are planned to validate these findings in future work. Future work will focus on the microfabrication of the proposed beam–island diaphragm and experimental validation of the FEM simulation results, including sensitivity, linearity, and acoustic performance under high-SPL conditions.

## Figures and Tables

**Table 1:** Mesh convergence study of the optimized structure under the 170 dB SPL equivalent pressure of 6324 Pa.

Mesh Level	Minimum Element Size Near Beam Root Region (μm)	Peak Structural Strain, *ε*_peak_ (με)	Maximum Displacement, *w*_max_ (μm)	Maximum Von Mises Stress, *σ*_max_ (MPa)	Relative Change of *ε*_peak_ (%)
Coarse	8	548.21	1.4538	76.182	3.64
Medium	5	562.53	1.466	79.539	1.13
Fine	3	568.94	1.4698	81.402	—

**Table 2:** Estimated voltage sensitivity and output voltage of the proposed graphene piezoresistive MEMS microphone using a quarter-bridge readout.

Gauge Factor, *GF*	Bridge Configuration	Estimated Voltage Sensitivity (µV/Pa)	Estimated Output at 170 dB SPL (mV)
2	Quarter bridge	0.223	1.41
4	Quarter bridge	0.445	2.81
10	Quarter bridge	1.11	7.04

**Table 3:** First-order thermal noise-limited SNR estimation for the quarter-bridge readout. The calculation assumes *T* = 300 K, *B* = 20 kHz, *R* = 5 kΩ, and *V*_n,th_ = 1.29 µV rms.

Gauge Factor, *GF*	Voltage Sensitivity (µV/Pa)	Output at 170 dB SPL (mV)	Equivalent Input Pressure Noise (Pa)	Estimated SNR at 170 dB SPL (dB)
2	0.223	1.41	5.79	60.8
4	0.445	2.81	2.90	66.8
10	1.11	7.04	1.16	74.8

**Table 4:** Comparison with representative high-SPL MEMS and graphene-based microphones. Reported sensitivities follow the convention of each original source. Values given as μV/V/Pa are normalized to the bias voltage, whereas values given as μV/Pa are absolute. When certain metrics, diaphragm dimensions, or fabrication details were not consistently reported in the original studies, they are listed as “Not reported” or summarized qualitatively in the positioning column.

Work	Mechanism/Structure	SPL/Dynamic Range	Sensitivity/Output/Bandwidth	Positioning/Fabrication Relevance
Sheplak et al. [3]	Silicon piezoresistive MEMS microphone	Linear response up to 155 dB SPL	~1 μV/V/Pa	Aeroacoustic MEMS benchmark; conventional silicon piezoresistive process; SPL below the 170 dB target
Martin et al. [4]	Dual-backplate capacitive MEMS microphone	164 dB SPL; dynamic range from a 41 dB/√Hz noise floor	390 μV/Pa; ~178 kHz resonance	High-SPL capacitive benchmark; backplate/air-gap structure may introduce gap-related nonlinearity and overload constraints
Yan et al. [5]	AlScN piezoelectric bimorph microphone	AOP 147 dB SPL; dynamic range 107.5 dB	Not directly comparable	Reliable piezoelectric readout; multilayer piezoelectric fabrication; AOP below the 170 dB target
Zhou et al. [15]	Wide-band piezoresistive MEMS aeroacoustic microphone	Not reported	~0.4 μV/V/Pa; resonance around 400 kHz	High-frequency aeroacoustic response; conventional piezoresistive readout
Ni et al. [9]	Graphene Fabry–Pérot optical acoustic sensor	Tested up to 114 dB SPL	5 Hz–0.8 MHz	Ultra-wide bandwidth; optical cavity readout and cavity packaging required
Zhao et al. [10]	Freestanding reduced-graphene-oxide membrane microphone	SNR up to 115 dB	100 Hz–50 kHz	High sensitivity; large compliant membrane; not focused on compact high-SPL MEMS robustness
Abrahams et al. [11]	Graphene squeeze-film microphone	Not reported	Resonance frequency modulation pressure readout	Compact graphene acoustic route; resonance-based output rather than flat broadband piezoresistive readout
This work	Graphene piezoresistive MEMS with membrane-supported beam–island diaphragm	170 dB reference pressure	Estimated quarter-bridge sensitivity 0.223–1.11 μV/Pa (normalized ≈ 0.045–0.22 μV/V/Pa at *V*_in_ = 5 V); 0–20 kHz variation ~0.94 dB; f_1_ = 64.07 kHz	1800 μm membrane side length; high-SPL strain localization with controlled deflection; simulation-stage design requiring graphene transfer, contact optimization, packaging, and acoustic validation

**Table 5:** Relative changes in key responses under ±5% fabrication deviation of each geometric parameter, with respect to the selected design.

Case	Δ*ε*_peak_ (%)	Δ*w*_max_ (%)	Δ*σ*_max_ (%)	Δf1 (%)
*t*_mem_ −5% (7.6 μm)	+4.16	+5.25	+3.91	−1.40
*t*_mem_ +5% (8.4 μm)	−3.78	−4.97	−3.69	+1.44
*t*_island_ −5% (21.85 μm)	+6.05	+10.05	+6.19	−3.38
*t*_island_ +5% (24.15 μm)	−5.91	−9.13	−6.25	+3.54
*L*_island_ −5% (475 μm)	+0.75	+2.01	+1.43	+0.52
*L*_island_ +5% (525 μm)	−0.68	−2.05	−0.59	−0.45
*L*_mem_ −5% (1710 μm)	−11.70	−15.68	−11.73	+14.05
*L*_mem_ +5% (1890 μm)	+18.19	+36.65	+18.37	−7.77

## Data Availability

The original contributions presented in this study are included in the article. Further inquiries can be directed to the corresponding authors.

## Abbreviations

The following abbreviations are used in this manuscript:

MEMS	Microelectromechanical systems
SPL	Sound pressure level
FEM	Finite-element method
